# Suppressed vascular Rho-kinase activation is a protective cardiovascular mechanism in obese female mice

**DOI:** 10.1042/BSR20230672

**Published:** 2023-07-17

**Authors:** Gabriela S. Barbosa, Rafael Menezes Costa, Wanessa M.C. Awata, Shubhnita Singh, Juliano V. Alves, Ariane Bruder-Nascimento, Camila R. Corrêa, Thiago Bruder-Nascimento

**Affiliations:** 1 Department of Pediatrics, UPMC Children’s Hospital of Pittsburgh at University of Pittsburgh, Pittsburgh, PA, U.S.A.; 2 Center for Pediatrics Research in Obesity and Metabolism (CPROM), UPMC Children’s Hospital of Pittsburgh at University of Pittsburgh, Pittsburgh, PA, U.S.A.; 3 UNIPEX, Medical School, São Paulo State University (UNESP), Botucatu, Brazil; 4 Endocrinology Division at UPMC Children’s Hospital of Pittsburgh, Pittsburgh, PA, U.S.A.; 5 Vascular Medicine Institute (VMI), University of Pittsburgh, Pittsburgh, PA, U.S.A.

**Keywords:** metabolic syndromes, obesity, vascular biology, vascular function, vascular smooth muscle

## Abstract

**Background:** Obesity is the number one cardiovascular risk factor for both men and women and is a complex condition. Although a sex dimorphism on vascular function has already been noted, the underlying processes remain unclear. The Rho-kinase pathway has a unique role in controlling vascular tone, and in obese male mice, hyperactivation of this system results in worsened vascular constriction. We investigated whether female mice exhibit decreased Rho-kinase activation as a protective mechanism in obesity.

**Methods:** We exposed male and female mice to a high-fat diet (HFD) for 14 weeks. At the end, energy expenditure, glucose tolerance, adipose tissue inflammation, and vascular function were investigated.

**Results:** Male mice were more sensitive to HFD-induced body weight gain, glucose tolerance, and inflammation than female mice. After establishing obesity, female mice demonstrated increase in energy expenditure, characterized by an increase in heat, whereas male mice did not. Interestingly, obese female mice, but not male, displayed attenuated vascular contractility to different agonists, such difference was blunted by inhibition of Rho-kinase, which was accompanied by a suppressed Rho-kinase activation, measured by Western blot. Finally, aortae from obese male mice displayed an exacerbated inflammation, whereas obese female demonstrated a mild vascular inflammation.

**Conclusion:** In obesity, female mice demonstrate a vascular protective mechanism—suppression of vascular Rho-kinase—to minimize the cardiovascular risk associated with obesity, whereas male mice do not generate any adaptive response. Future investigations can help to understand how Rho-kinase becomes suppressed in female during obesity.

## Introduction

Obesity is a multifactorial disease with a complex pathogenesis associated with psychosocial socioeconomic, biological, and environmental mechanisms [1]. Obesity is a major public health issue that has been on the rise in the world. The World Health Organization (WHO) reports that the global obesity rate has nearly tripled since 1975, and the obesity epidemic is now well-established [2]. Obesity increases the risk of a number of metabolic abnormalities, including Type 2 diabetes, hypertension, inflammation, and dyslipidemia, which are major risk factors of vascular injury and cardiovascular disease (CVD) [1–3]. Therefore, to enhance the quality of life and lower the mortality linked to this condition, it is essential to understand the vascular pathways causing CVD in obesity.

Obesity is a leading risk factor for CVD and a major health burden in male and female [4–6]. However, the sex-discrepant mechanism is implicated in obesity-associated CVD [7–9]. Sex hormones, as well as sex chromosomes themselves can cooperate to the development of obesity, glucose metabolism, and vascular function regulation [5–11]. In 2017 [5], we showed that females display a slower body weight gain compared to male mice under high-fat diet (HFD), which is followed by protection against obesity-induced sympathetic activation and changes in adrenergic vascular contractility. Although we observed a difference in vascular response in male and female mice, we did not identify the underlying vascular mechanisms.

Rho-kinase, a downstream target in the RhoA-linked pathway, is formerly identified as an effector of the small GTPase Rho. In the vasculature, RhoA-linked pathway can determine motility, morphology, polarity, cell division, gene expression, and cellular contraction. Rho-kinase promotes vascular contraction via a complex and extensive network between RhoA, Rho-kinase or ROCK (ROCKα/ROCK2), ROCKβ/ROCK1, myosin phosphatase target subunit 1 (MYPT1), and myosin light chain (MLC) [12–14]. Increased vascular tone in obesity has been previously demonstrated to be Rho-kinase overactivation-dependent in males [15,16], but whether females present the same Rho-kinase overactivation dependence on vascular contraction in obesity is still to be determined.

In addition to characterizing the body weight gain, glucose sensitivity, and energy expenditure in male and female mice under HFD treatment, the present study also sought to understand the difference in vascular contractility between male and female mice in obesity. Therefore, we tested the hypothesis that female mice display a suppressed Rho-kinase activation as a compensatory mechanism to attenuate vascular contractility in obesity.

## Methods

### Mice

Male and female C57Bl/6 mice (6–8 weeks of age) were divided into four groups and fed either a normal diet (ND; Research Diets, D12328, Carbohydrate 73%, Fat 11%, and Protein 16% kcal) or a high-fat diet (HFD; Research Diets, D12492, 60% of fat calories; 20% of protein and 20% of carbohydrate) ad libitum. Tap water was provided ad libitum. Mice were monitored for 14 weeks. Body weight was measured weekly. At the end of the experiments, mice were euthanized by carbon dioxide (CO_2_) asphyxiation, then gonadal, retroperitoneal, visceral, subcutaneous, subscapular brown adipose tissue, heart, liver, and kidneys were isolated and weighed for adiposity and cardio-renal characterization.

### Energy expenditure

The Oxymax Lab Animal Monitoring System (CLAMS, Columbus Instruments, Columbus, OH) was used to determine heat and respiratory exchange ratio (RER) calculated from CO_2_ production and O_2_ uptake ratio as described before [17]. Mice were placed on CLAMS for 2 days of acclimatization, then the parameters mentioned above were recorded for 72 h. Area under curve from 72 h record was used to determine any difference.

### Intraperitoneal glucose tolerance test (ipGTT)

Intraperitoneal glucose tolerance test (ipGTT) was performed to evaluate glucose intolerance. Mice were deprived of food for 12 h. Blood sample was collected from the caudal vein immediately before (baseline, *t*_0_) and after (*t*_15_, *t*_30_, *t*_60_, *t*_90_, *t*_120_ min) administration of 2 g of glucose/kg by intraperitoneal injection. Glucose levels were determined using a glucose analyzer (Accu-Check, Roche Diagnostics) as previously described [18].

### Vascular remodeling

Mice were euthanized for aortae harvest and perfused with cold phosphate-buffered saline (PBS). Aortae were collected and placed in a 4% paraformaldehyde (PFA) solution for histology analysis. After 12 h in PFA, tissues were placed in 70% ethanol until the day of preparing the samples for histology. Aortae were embedded in paraffin, then samples were sectioned and stained with hematoxylin and eosin (H&E) to analyze the vascular remodeling and structure.

### Adipose tissue and vascular inflammation

mRNA from gonadal fat and aorta were extracted using RNeasy Mini Kit (Quiagen, Germantown, MD, U.S.A.). Complementary DNA (cDNA) was generated by reverse transcription polymerase chain reaction (RT-PCR) with SuperScript III (Thermo Fisher Waltham, MA U.S.A.). Reverse transcription was performed at 58°C for 50 min; the enzyme was heat inactivated at 85°C for 5 min, and real-time quantitative RT-PCR was performed with the PowerTrackTM SYBR Green Master Mix (Thermo Fisher, Waltham, MA U.S.A.). Sequences of genes as listed in Table 1. Experiments were performed in a QuantStudioTM 5 Real-Time PCR System, 384-well (Thermo Fisher, Waltham, MA U.S.A.). Data were quantified by 2ΔΔ *C*t and are presented by fold changes indicative of either up-regulation or down-regulation.

**Table 1:** List of primers

Primer	Sequence	
CCR1	FW	GCCAAAAGACTGCTGTAAGAGCC
	RV	GCTTTGAAGCCTCCTATGCTGC
CCR3	FW	CCACTGTACTCCCTGGTGTTCA
	RV	GGACAGTGAAGAGAAAGAGCAGG
CCR5	FW	GGTTCCTGAAAGCGGCTGTAAATA
	RV	CTGTTGGCAGTCAGGCACATC
F4/80	FW	TCCTGCTGTGTCGTGCTGTTC
	RV	GCCGTCTGGTTGTCAGTCTTGTC
IL6	FW	TTCTTGGGACTGCTGGT
	RV	CAGGTCTGTTGGGAGTGGTA
TNFα	FW	AATGGCCTCCCTCTCATCAG
	RV	CCTAACTGCCCTTCCTCCAT
Ki67	FW	AGAGCCTTAGCAATAGCAACG
	RV	GTCTCCCGCGATTCCTCTG
VCAM1	FW	TGACAAGTCCCCATCGTTGA
	RV	ACCTCGCGACGGCATAATT
ICAM1	FW	ATCACATGGGTCGAGGGTTT
	RV	AACCACTGCCAGTCCACATA
GAPDH	FW	GAGAGGCCCTATCCCAACTC
	RV	TCAAGAGAGTAGGGAGGGCT

Primers were purchased from Integrated DNA Technologies.

### Western blot

Aortic protein was extracted using radioimmunoprecipitation assay buffer (RIPA) buffer (30 mM HEPES, pH 7.4, 150 mM NaCl, 1% Nonidet P-40, 0.5% sodium deoxycholate, 0.1% sodium dodecyl sulfate, 5 mM EDTA, 1 mM NaVO4, 50 mM NaF, 1 mM PMSF, 10% pepstatin A, 10 μg/ml leupeptin, and 10 μg/ml aprotinin). Protein samples were suspended in Laemmli sample buffer supplemented with 2-mercaptoethanol (β-mercaptoethanol) (BioRad Hercules, California, U.S.A.). Then, proteins were separated by electrophoresis on a polyacrylamide gradient gel (BioRad Hercules, California, U.S.A.), and transferred to Immobilon-P poly (vinylidene fluoride) membranes. Non-specific binding sites were blocked with 5% skim milk or 1% bovine serum albumin (BSA) in tris-buffered saline solution with tween for 1 h at 24°C. Membranes were then incubated with specific antibodies overnight at 4°C as described in Table 2. After incubation with secondary antibodies (BioRad Hercules, California, U.S.A.), the enhanced chemiluminescence luminol reagent (SuperSignalTM West Femto Maximum Sensitivity Substrate, Thermo Fisher Waltham, MA, U.S.A.) was used for antibody detection.

**Table 2:** List of antibodies

Antibody	Catalog number	Company	Concentration
α-SMA	19245	Cell Signaling	1:2000
Thr^853^ Mypt1	4563	Cell Signaling	1:500
RhoA	2117	Cell Signaling	1:1000
ROCK1	28999	Cell Signaling	1:1000
ROCK2	47012	Cell Signaling	1:1000
β-Actin	A3854	Sigma	1:20000

### Vascular reactivity

Endothelium intact aortic rings were mounted in a wire myograph (Danysh MyoTechnology) for isometric tension recordings with PowerLab software (AD Instruments) as described [18–21]. Briefly, rings (2 mm) were placed in tissue baths containing warmed (37°C), aerated (95% O_2_, 5% CO_2_) Krebs Henseleit Solution: (in mM: 130 NaCl, 4.7 KCl, 1.17 MgSO_4_, 0.03 EDTA, 1.6 CaCl_2_, 14.9 NaHCO_3_, 1.18 KH_2_PO_4_, and 5.5 glucose) and after 30 min of stabilization, arteries were incubated with KCl (60 mM) to test the sample viability. Then, the following concentration response curves (CRC) were performed: Phenylephrine and thromboxane analogue (U46619). To study the role of Rho-Kinase pathway, we inhibited ROCK with Y-27632 (100 µM) and performed CRC to U46619f.

### Statistical analysis

Our aim was to determine the impact of HFD on male and female mice, thus we used Student’s *t*-test to determine any difference between ND and HFD in both sexes. The vascular contractility data are expressed in millinewton (mN). The concentration-response curves were fitted by nonlinear regression analysis. Maximal response (*E*_max_) was determined. Analyses were performed using Prism 9.0 software (GraphPad). A difference was considered statistically significant when *P*≤0.05.

## Results

### Female mice present an attenuated body weight gain, energy expenditure impairment, and glucose tolerance in HFD-induced obesity model

**Figure 1:**
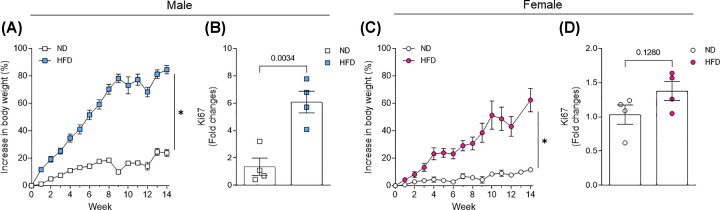
Body weight gain in male and female mice under HFD treatment Body weight gain (**A,C**) and gene proliferation marker in gonadal fat (Ki67, **B,D**) from male and female mice exposed to HFD for 14 weeks. Body weight was analyzed weekly. Ki67 expression was analyzed by RT-PCR. Data are presented as mean ± standard error of the mean (SEM). *N* = 4 for RT-PCR and 8 for body weight gain. *P<0.05 vs. ND.

First, we investigated if HFD promotes obesity and impairs energy expenditure and glucose sensitive in male and female mice. By measuring body weight gain and fresh weight of different adipose tissue depots (Figure 1A,C and Table 3), we observed that HFD induced obesity in male and female, however female mice were more resistance to obesity appearance. Such an increase in adiposity was followed by a significant enhancement in Ki67 (a marker of proliferation) in gonadal fat in males but not in females (Figure 1B,D).

**Table 3:** Characterization of adiposity and cardiorenal system of male and female mice exposed to normal diet or high-fat diet

Variable	Groups			
	Male ND	Male HFD	Female ND	Female HFD
Initial body mass (g)	24.9 ± 0.9	26.1 ± 0.42	22.9 ± 0.3	19.2 ± 0.4
Final body mass (g)	30.9 ± 1.36	48.2 ± 0.9*	22.8 ± 0.3	30.7 ± 2.0^#^
Weight gain (g)	6.0 ± 0.71	22.1 ± 0.8*	1.9 ± 0.3	11.5 ± 1.7^#^
Gonadal adipose tissue (g)	0.25 ± 0.02	1.07 ± 0.09*	0.20 ± 0.03	0.86 ± 0.19^#^
Retroperitoneal adipose tissue (g)	0.08 ± 0.01	1.09 ± 0.04*	0.07 ± 0.01	0.59 ± 0.12^#^
Visceral adipose tissue (g)	0.12 ± 0.03	1.01 ± 0.11*	0.11 ± 0.01	0.33 ± 0.07^#^
Subcutaneous adipose tissue (g)	0.30 ± 0.05	2.09 ± 0.07*	0.26 ± 0.02*	1.01 ± 0.16^#^
Subscapular brown adipose tissue (g)	0.07 ± 0.01	0.23 ± 0.01*	0.10 ± 0.01	0.12 ± 0.01
Adiposity index (%)	2.29 ± 0.26	11.23 ± 0.73*	2.87 ± 0.20	8.63 ± 1.16^#^
Heart (g)	0.08 ± 0.01	0.10 ± 0.01*	0.07 ± 0.01	0.10 ± 0.02^#^
Liver (g)	0.90 ± 0.04	1.80 ± 0.06*	0.65 ± 0.02	0.81 ± 0.05^#^
Kidney (g)	0.24 ± 0.01	0.30 ± 0.02*	0.17 ± 0.01	0.21 ± 0.01^#^

Data are presented as Mean ± SEM; *N* = 4–8. *P<0.05 vs. male ND; ^#^*P*<0.05 vs. female ND. Statistic analyzed was performed by comparing ND and HFD within the same sex.

**Figure 2:**
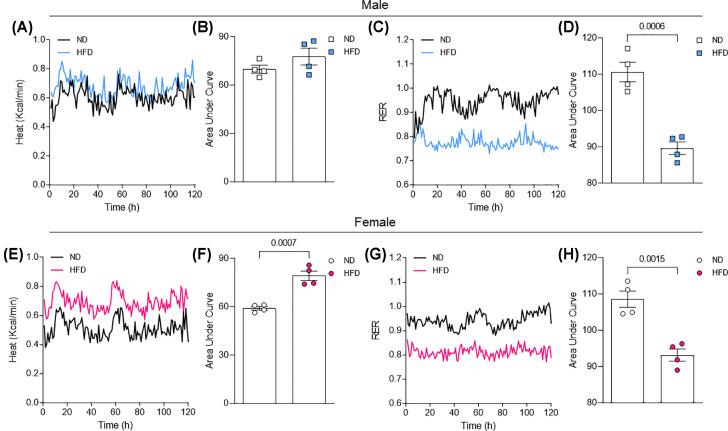
Energy expenditure in male and female mice exposed to HFD Heat (**A,B,E,F**) and respiratory exchange ratio (RER) (**C,D,G,H**) from male and female mice exposed to ND or HFD for 11 weeks. Area under curve data are presented as mean ± standard error of the mean (SEM). *N*=4; *P<0.05 vs. ND.

Furthermore, we observed that male mice do not present increase in heat after HFD treatment (Figure 2A,B), different from female mice, which demonstrated elevated heat post HFD treatment (Figure 2E,F), indicating that differences in heat generation may in fact be associated with the severity of obesity in males and females. Finally, HFD exposure decreased RER in male and female mice (Figure 2C,D,G,H).

**Figure 3:**
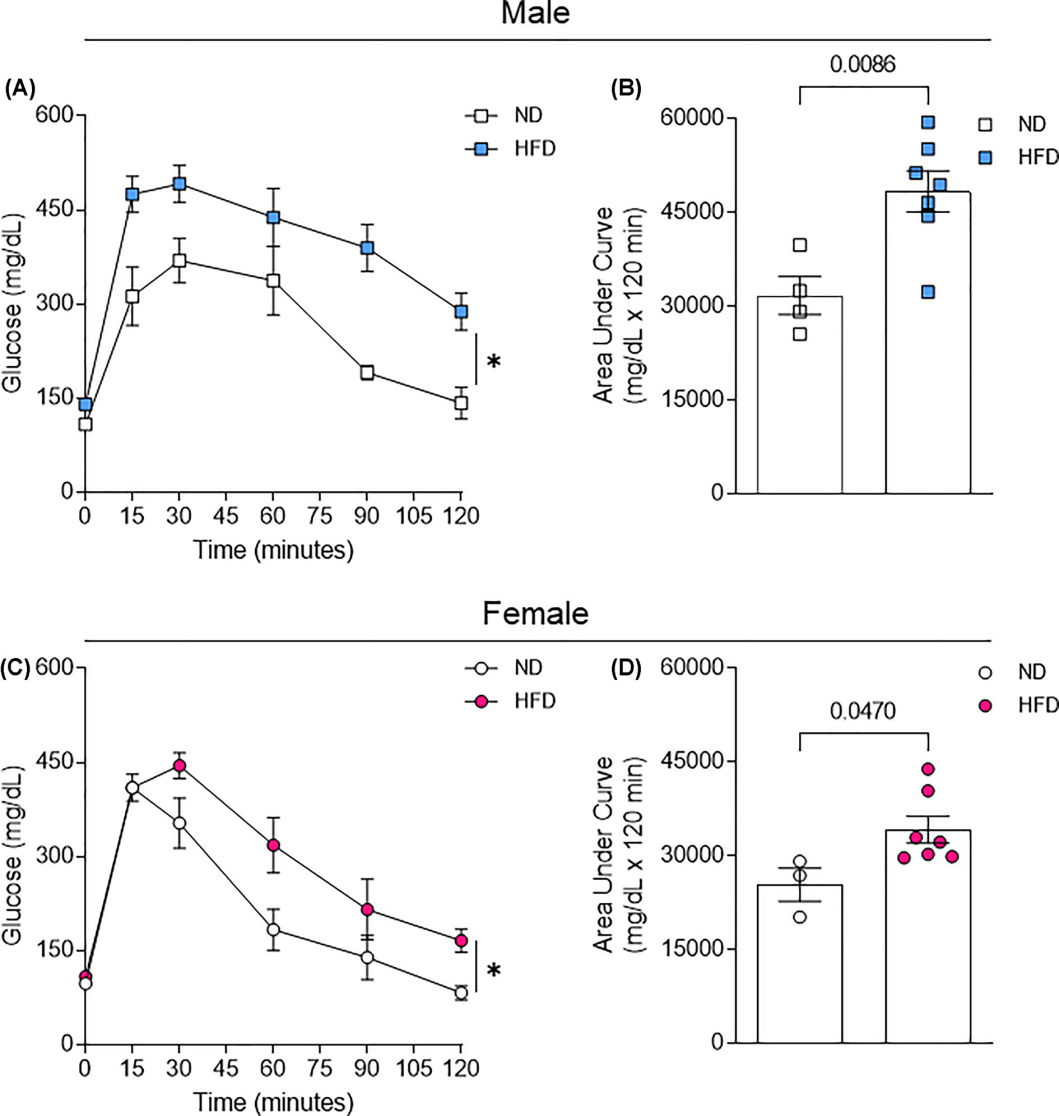
Glucose tolerance in male and female mice exposed to HFD Intraperitoneal glucose intolerance test (ipGTT) in male (**A,B**) and female (**C,D**) mice exposed to normal diet (ND) or HFD for 11 weeks. Data are presented as mean ± standard error of the mean (SEM). N = 4. *P<0.05 vs. ND.

Since obesity is associated with glucose intolerance, we investigated how is the glucose sensitive in male and female mice under HFD treatment, we found that HFD induced glucose intolerance in male and female mice, but male mice appeared to be more resistant to HFD-induced glucose intolerance. (Figure 3A–D).

### Female mice are resistant to HFD-induced obesity-associated adipose tissue inflammation

**Figure 4:**
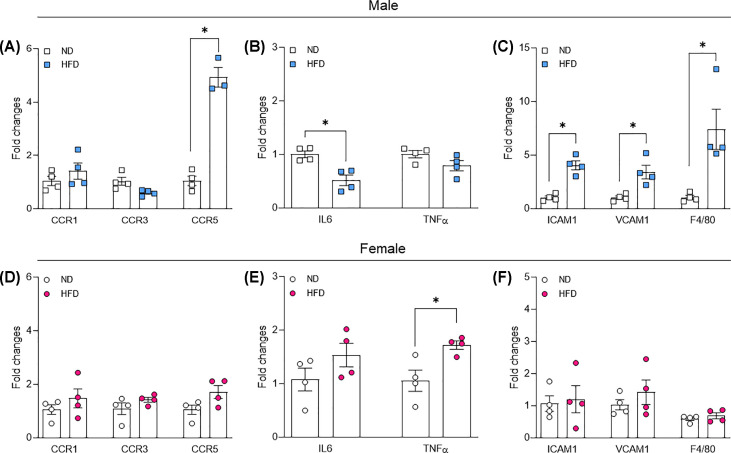
Adipose tissue inflammation in male and female mice exposed to HFD Chemokines expression (**A,D**), cytokines (**B,E**), and adhesion gene and macrophage marker (F4/80) expression (**C,F**) in gonadal fat from male and female mice exposed to normal diet (ND) or HFD for 14 weeks. Gene expression was analyzed by RT-PCR. Data are presented as mean ± standard error of the mean (SEM); *N*=4. *P<0.05 vs. ND.

Low-grade inflammation of adipose tissue is a key characteristic of obesity. We investigated, via RT-PCR, the inflammation level in gonadal fat from male and female mice and found that CCR5, ICAM1, VCAM1, and F4/80 (macrophage marker) are elevated only in gonadal fat from males exposed to HFD, whereas TNFα was elevated only in female treated with HFD. Finally, IL6 gene expression was surprisingly decreased in gonadal fat from males treated with HFD (Figure 4A–F).

### Obese female mice demonstrate attenuated vascular contractility with no changes in vascular hypertrophy or contractile protein

**Figure 5:**
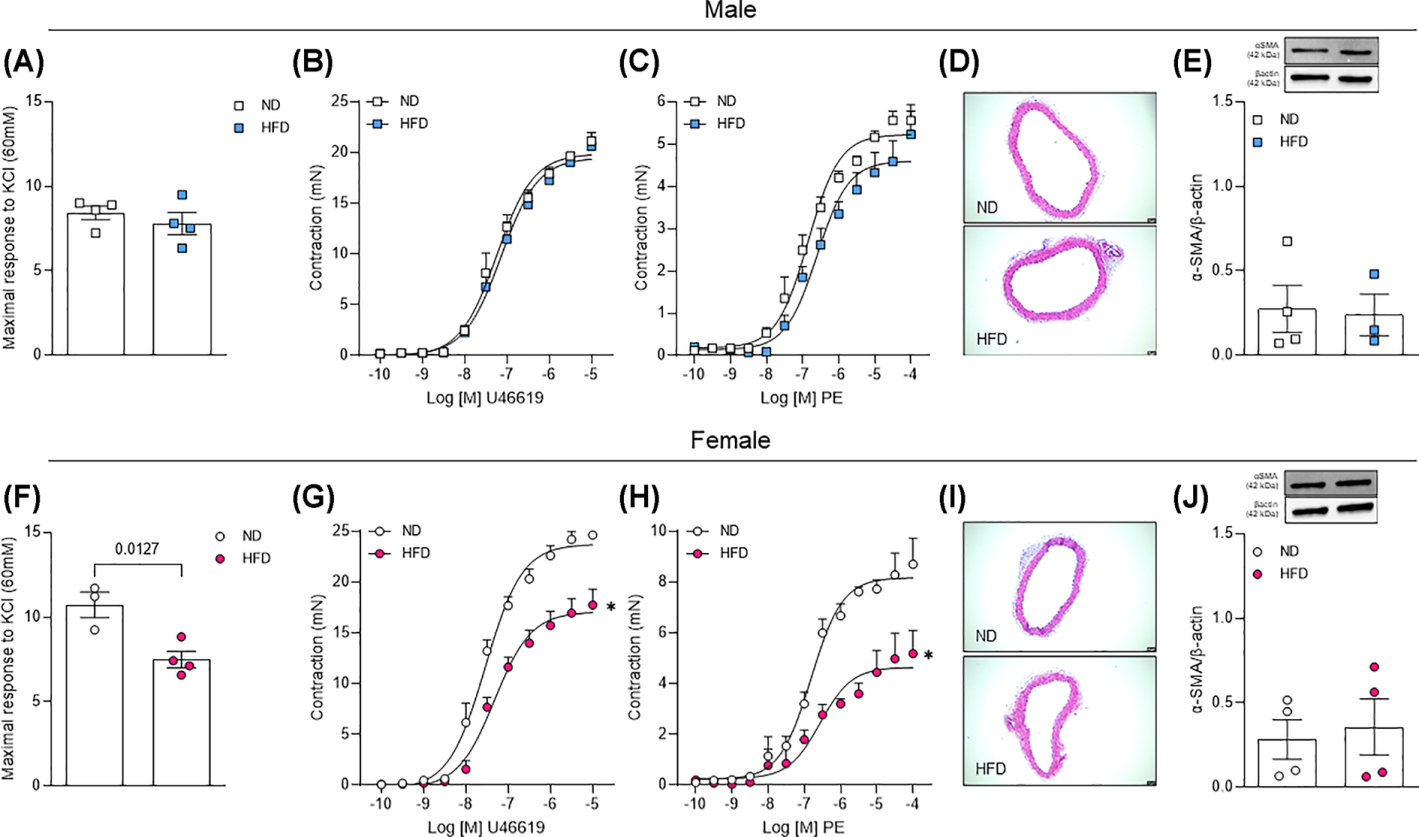
Vascular function and structure from male and female mice exposed to HFD KCl, 60 mM response (**A,F**) and CRC to thromboxane analogue, U46619 (**B,G**) or phenylephrine (**C,H**) in endothelium intact aortic rings. Aortic remodeling (**D,I**) and aortic smooth muscle α-SMA (**E,J**) expression. Experiments were performed in vascular samples from male and female mice exposed to ND or HFD for 14 weeks. Data are presented as mean ± standard error of the mean (SEM); *N*=4; *P<0.05 vs. ND.

Interestingly HFD treatment did not affect the vascular contractility in male mice analyzed by KCl, thromboxane analogue, and phenylephrine responses (Figure5A–C); however, female mice demonstrated an attenuated vascular contraction to KCl, thromboxane analogue, and phenylephrine (Figure 5F–H). Finally, changes in vascular response were not dependent on structural modifications or contractile protein content, measured by H&E staining and α-actin (α-SMA) amount, respectively (Figure 5D,E,I,J).

### Attenuated vascular contractility in obese female mice is mediated by a suppressed Rho-kinase activity

**Figure 6:**
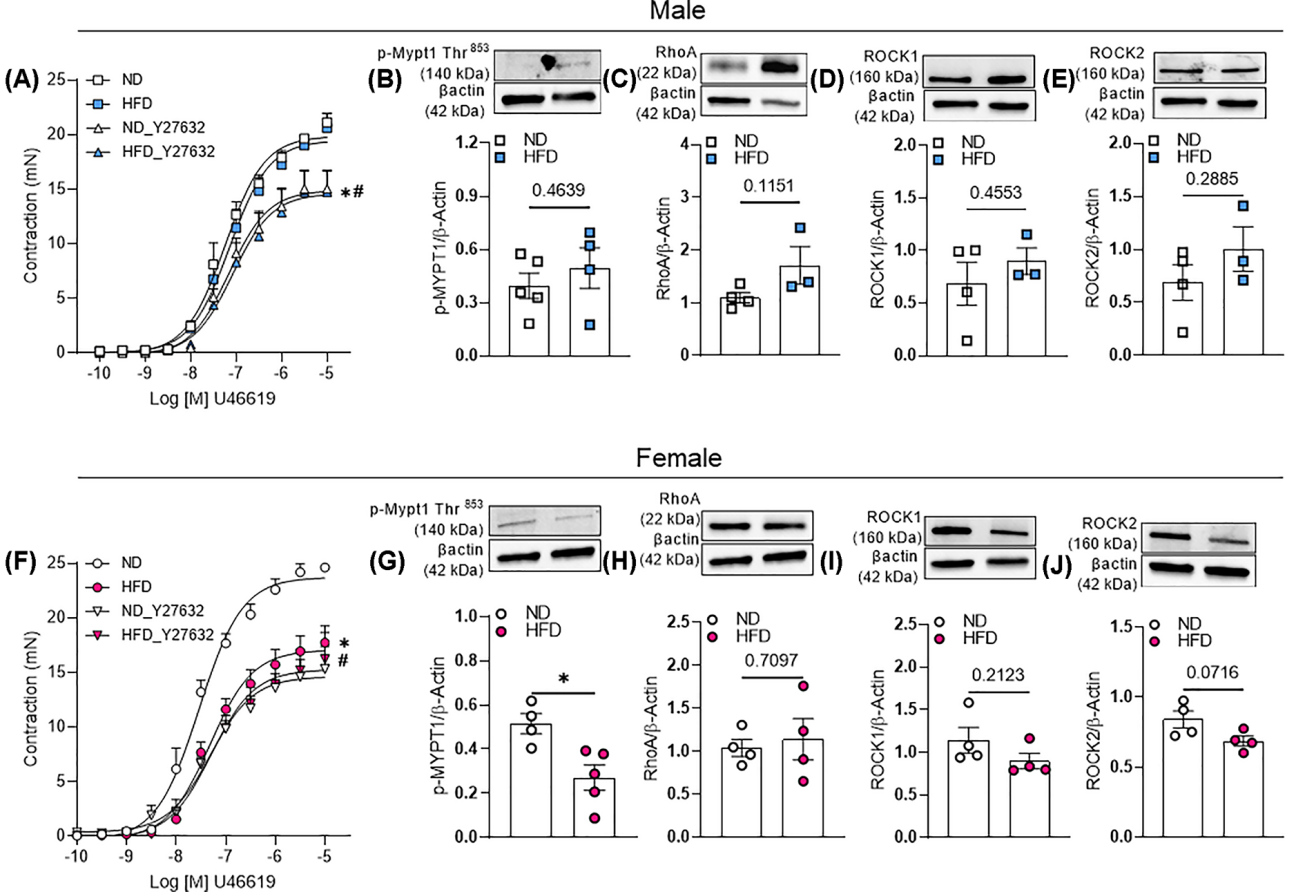
Role of Rho-kinase pathway on vascular dysfunction associated with HFD treatment CRC to thromboxane analogue, U46619 with or without ROCK inhibitor (**A,F**) in endothelium intact aortic rings. Expression of Rho-kinase pathway-associated proteins in aortae (**B–E,G–J**) analyzed by western blot. Experiments were performed in vascular samples from male and female mice exposed to ND or HFD for 14 weeks. Data are presented as mean ± standard error of the mean (SEM). *N*=4. *P<0.05 vs. ND; ^#^*P*<0.05 vs. without Y27632.

To study by which mechanism obese female mice display attenuated vascular contractility we inhibited Rho-kinase pathway via Y-27632. We observed that Y-27632 similarly affected the vascular contractility in arteries from lean and obese male mice (Figure 6A). Furthermore, no difference in Mypt1 phosphorylation or total RhoA, ROCK1 and 2 was found in arteries from lean and obese male mice (Figure 6B–E).

**Figure 7:**
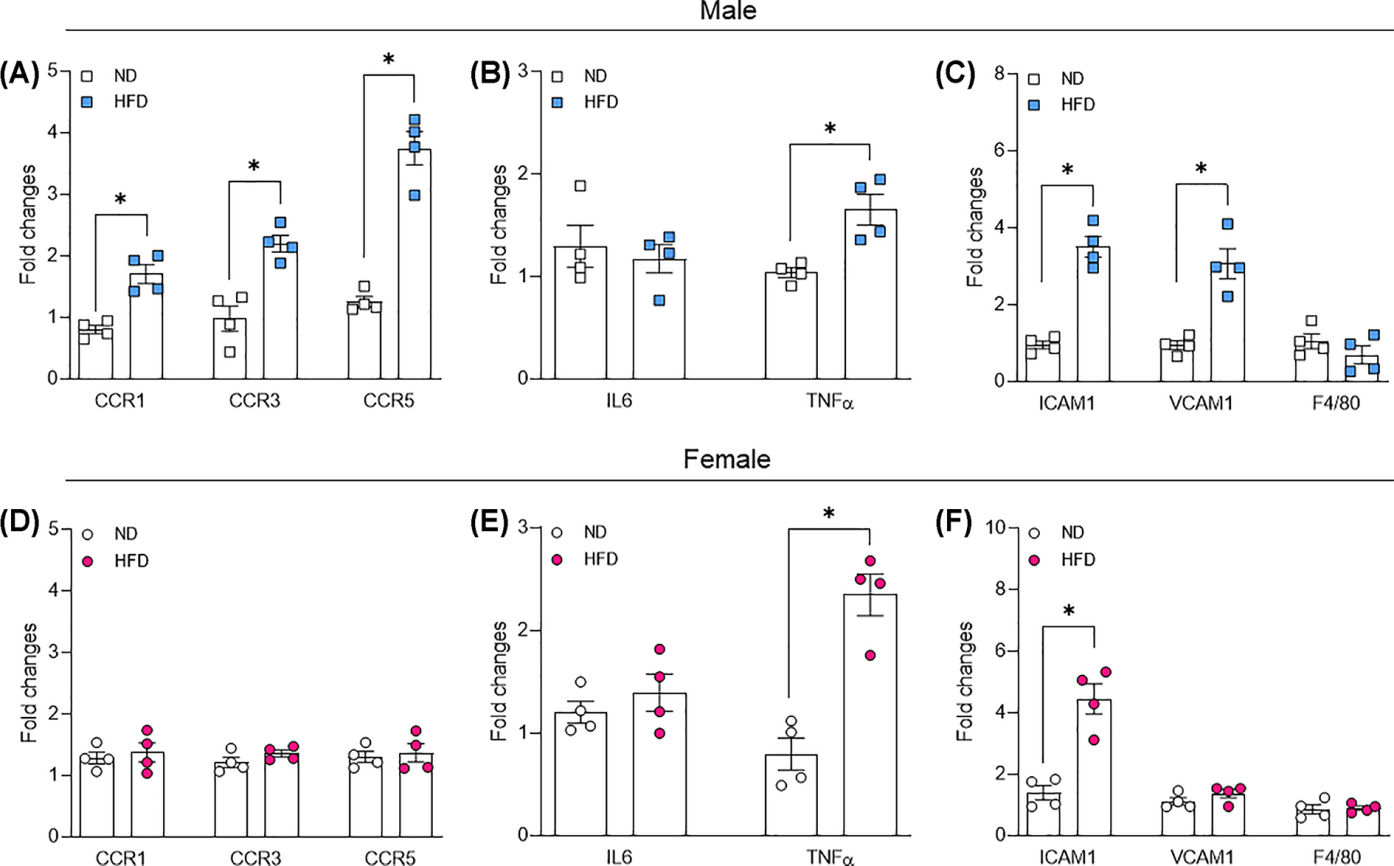
Obese male mice display an exacerbated vascular inflammation Chemokines (**A,D**), cytokines (**B,E**), and adhesion gene and macrophage marker (F4/80) expression (**C,F**) in aortae from male and female mice exposed to ND or HFD for 14 weeks. Gene expression was analyzed by RT-PCR. Data are presented as mean ± standard error of the mean (SEM); *N*=4; *P<0.05 vs. ND.

We also observed that Y-27632 only affected the response of arteries from lean female mice but not obese female mice (Figure 6F), suggesting that Rho-kinase pathway is attenuated in female mice exposed to HFD. Finally, decreased phosphorylated Mypt1 at Thr^853^ residue, which is involved in RhoA/ROCK-mediated inhibition of myosin phosphatase [12], was found in arteries from obese female mice (Figure 6G), further confirming decreased Rho-kinase pathway. No difference was found for RhoA and ROCK1 and2 expression (Figure 6H–J). Finally, Rho-kinase is a redox and inflammatory sensitive protein [15], thus, we investigated the vascular inflammatory profile in in our different groups. We interestingly found that the expression of chemotactic markers, inflammatory cytokines, and adhesion molecules are increased in the aorta of obese males (Figure 7A–C), whereas only TNFα and ICAM1 are increased in aortas of obese females (Figure 7D–F).

## Discussion

In the present study, we sought to describe the sex-specificity of the mechanisms controlling vascular contractility in obesity and pinpoint the source of any potential sex-discrepancy. Also, we investigated how body weight, metabolic issues, energy usage, and inflammation might be linked to vascular dysfunction. Our key findings are: (1) male and female mice develop characteristics of obesity HFD, but female mice are more resistant to HFD-induced body weight gain; (2) female mice present a better energy expenditure behavior under HFD; (3) aortae from obese female displayed hypocontractility, whereas male mice do not demonstrate any alteration; (4) finally female exposed to HFD show suppressed Rho-kinase activity. In light of these findings, we first established that female mice have lower Rho-kinase activation throughout the development of obesity as a potential compensatory strategy to safeguard the vasculature against obesity-related vascular damage.

HFD in rodents induces a sexual dimorphism in body weight, metabolic alterations, and degree of inflammation [22–26]. Female mice are commonly leaner and exhibit reduced increases in body weight, preserved metabolic function, and lower degree of inflammation as compared with male [24–27]. We previously demonstrated that male mice display a significant increase in body weight under obesogenic diet from week 3, whereas female only after week 9. In the same study, we observed that differences between male and female mice disappear only after 18 weeks of HFD intervention [5], indicating that female mice have a slower body weight gain under obesogenic diet, which might be associated with increased energy expenditure—since female demonstrated elevate heat in CLAMS analyze—therefore, an elevated energy burn could be a gatekeeper against the fat accumulation in female mice under obesogenic diet. Finally, we only treated our mice for 14 weeks with obesogenic diet, perhaps exposing the mice longer would blunt any sex difference at the end.

Fat accumulation can lead to impaired glucose response by promoting insulin resistance and disrupting glucose uptake and metabolism, which appears to be a sex-specific response. Male mice after 14 or 16 weeks of Western diet [22] or HFD [24,27] demonstrate a worse glucose metabolism compared with their female counterpart. On the other hand, male and female mice became glucose tolerant, but males were more sensitive to obesogenic diet. Inflammation of adipose tissue is a key precursor of glucose tolerance [28,29]; however, female mice tend to present a lower inflammation than male under HFD [24,30]. We observed that chemokine receptor CCR5, adhesion genes (VCAM1 and ICAM1), as well as F4/80 (macrophage marker) were up-regulated in gonadal fat from obese male in at least 4-fold increases, in contrast female only displayed a mild increase in TNFα. CCR5 plays a major role in controlling obesity-induced adipose tissue inflammation and insulin resistance by regulating macrophage recruitment [31], therefore increased sensitivity to HFD-induced inflammation in male, likely dependent on CCR5 and macrophages, would justify why males become more glucose tolerant to HFD.

We and others have demonstrated that obesity affects the function of large and small arteries [5,16,18,32–34] in a sex discrepancy-dependent manner [5,7,8,22]. Although such information is already well-established, the molecular mechanisms is not fully comprehended. In the present study, we observed that only aortae from female exposed to obesogenic diet presented attenuated response to different contractile agonists, which was not associated with changes in vascular remodeling or contractile protein amount indicating that an intracellular signaling is altered only in female. Therefore, we investigated an important signaling pathway associated with cardiovascular risks [16,35–38], the Rho-kinase pathway.

Multiple vascular contractile agonists generate their responses by activating Rho-kinase pathway including endothelin-1 [39], angiotensin-II [40], and arachidonic acid metabolites (thromboxane A_2_) [41], in common, these important mediators are elevated in obesity [42–45]. We found that (1) inhibiting Rho-kinase signaling blunted the difference on vascular contraction between lean and obese female and (2) Mypt-1 phosphorylation at Thr^853^ is attenuated in arteries from obese female mice, when taken together, these data imply that the Rho-kinase pathway is inhibited in the arteries of female mice given HFD. Many compensatory mechanisms in the vasculature of obese females have been proposed. Here, we propose for the first time that RhoA pathway blockage occurs to ensure a protection against obesity-induced cardiovascular risk in females. Previous findings suggest that estrogen suppresses Rho-kinase function in the cerebral circulation [46], induces neuroprotective effects of in model of Parkinson’s disease via inhibiting Rho-kinase [47], and causes a decrease in Rho-kinase mRNA expression [48]. Thus, we can suggest that increase in estrogen might be regulating the suppression of Rho-kinase pathway in obese female. Further investigations are necessary to confirm whether changes in estrogen signaling are driving the vascular protection in female mice and whether such adaptive response is endothelium dependent.

In summary, our data indicate that male mice are more susceptible to gain body weight compared with female mice, which is associated with impaired energy expenditure, higher glucose tolerance, and exacerbated adipose tissue inflammation. Our findings also suggest that female mice under obesogenic diet demonstrate a vascular protective effect by attenuating the Rho-kinase pathway, whereas male mice lack in any adaptive response.

Future studies will help to elucidate by which endocrine and vascular mechanisms female mice display such protection via Rho-kinase suppression. Overall, our data add one more piece to the literature that sex should be considered an important variable when identifying the adequate therapeutic strategy for treatment of obesity associated vascular dysfunction as the therapies which are effective in one sex may not be effective in other

## Data Availability

The data that support the findings of this study are available from the corresponding author on reasonable request. Western blot membranes without crops are available in the supplementary file.

## Abbreviations

α-SMA: α-smooth muscle actin
CVD: cardiovascular diseases
CRC: concentration response curves
H&E: hematoxylin and eosin
HFD: high-fat diet
ipGTT: intraperitoneal glucose intolerance test
MLC: myosin light chain
MYPT1: myosin phosphatase target subunit 1
ND: normal diet
PFA: paraformaldehyde
RER: respiratory exchange ratio
